# Experimental evaluation of soapberry seed oil biodiesel performance in CRDI diesel engine

**DOI:** 10.1038/s41598-023-32424-8

**Published:** 2023-04-07

**Authors:** Mohammed Owais Ahmed Sajjad, T. Sathish, M. Rajasimman, T. R. Praveenkumar

**Affiliations:** 1 Department of Mechanical Engineering, Saveetha School of Engineeering, SIMATS, Chennai, Tamil Nadu India; 2 Department of Chemical Engineering, Annamalai University, Annamalai Nagar, 608002 India; 3 Department of Construction Technology and Management, Wollega University, Western Oromia, Ethiopia

**Keywords:** Mechanical engineering, Biodiesel

## Abstract

Due to the ongoing demand for alternative fuels for CI engines, biodiesel-based research has received support globally. In this study, soapberry seed oil produced by transesterification process to creates biodiesel. It is referred to as BDSS (Biodiesel of Soapberry Seed). According to criteria, the oil qualities are recognized, hence, three different blends and pure diesel were tested in CRDI (Common Rail Direct Injection) engines. The blends descriptions are: 10BDSS (10% BDSS + 90% diesel), 20BDSS (20% BDSS + 80% diesel), and 30BDSS (30% BDSS + 70% diesel). The outcomes of the related tests for combustion, performance, and pollution were contrasted with those achieved using 100% diesel fuel. In this case, the mixing has resulted in worse braking thermal efficiency than diesel and lower residual emissions with greater NOx emissions. The superior results were obtained by 30BDSS, which had BTE of 27.82%, NOx emissions of 1348 ppm, peak pressure of 78.93 bar, heat release rate (HRR) of 61.15 J/deg, emissions of CO (0.81%), HC (11 ppm), and smoke opacity of 15.38%.

## Introduction

Alternate fuels from the natural sources based research is the most concentrated area of the current fossil fuel demand criteria. The traditional fuel resources get going down as well as they produced more emission to the environment, which are leads to harmful effects to the living organisms and affect the environment feasibility^1^. There are so many biodiesel can be produced form the different kind of seeds of eatable and non-eatable vegetable oils. Also so many biodiesel also produced with waste products in nature and artificial.

Venkatesan et al.^2^ mentioned that the particulate matters and NOx emission production by the heavy load CI engine (Construction, loading, agricultural machineries) will be increased in 2035 itself as NOx and PM have 70% and 85% increase. Over inhaling of PM create death to premature livings^3^. Jayabal et al.^4^ mentioned that the smoke opacity produced from the CI engine is injurious to livings, which cause cardiac diseases, breathing problems.

The biodiesel is the one of the fuel used in the CI engine. There are so many studies carried by different researches in verities of the biomaterials with and without diesel. There different methods available for the preparation of the biodiesel^5^. Among that transesterification is one the easiest and best method to extract the biodiesel^6^.

Zimmerman et al.^7^ clearly explained about the esterification process based challenges and methods to overcome those problems. Muthukumaran et al.^8^ deals with the biodiesel production from the oil of Madhuca Indica by transesterification process. They mentioned that potassium hydrazide have better influence on the biodiesel production process. Oil at 60 °C for one and half an hour treatment with methanol (0.32%) with catalyst (1.5%) produce 51% of biodiesel yield.

Moradi et al.^9^ deals with the influencing factors on production of biodiesel from the castor seeds. They practice in the Soxhlet extractor. The solvents used for the oil extraction are acetone and methanol. 75 °C temperature mainlining for six hours produced better results on the yield. Kamil et al.^10^ deals with the biodiesel blends of turkey lard and swine lard in CRDI engine. Diesel blend with methyl ester produced lesser carbon monoxide (20%), carbon di oxide (6%) and hydrocarbon emission (9%).

Alptekin et al.^11^ deals with methyl and ethyl esters of the vegetable oil fueled in CRDI engine with various loads. From the experimental results, it is clearly mentioned that the fuels considered have lesser peak pressure, more heat release rate with higher fuel consumption than the diesel fuel. The timing of pilot and main injection were higher than the diesel injection timing. Yingqun et al.^12^ deals with the biodiesel of palm oil in CRDI engine with different injection pressure. The increase on the fuel injection pressure increase the NOx emission and reduce the PM, CO, and HC emission at higher loads.

Ogunkunle et al.^13^ deals with biodiesel of Parinari polyandra in CI engine. They compare the different blends among that 10% biodiesel blend produce augmented results on the thermal efficiency and power. It produce more NOx emission but the remaining emissions were lesser than diesel. 81.7% of CO_2_ and 65.7% of CO reduction than diesel, obtained with 10% of biodiesel blend.

Pavan et al.^14^ deals with the methyl esters of palm oil in CRDI engine with different injection pressure with splits. 40% NOx reduction can be achieved by the 23-degree before TDC single injection as well as 32 degree before TDC higher injection with pilot injection (10%)0.26.2% of CO, 19.2% of HC and 21.5% of smoke opacity reduction can be achieved in early injection (34 degree before TDC).

There are numerous species (nearly thousand) are available in the Soapberry family. These are spread over the world such as some places in Australia and Africa majorly in South America and Asia. In china itself alone, Soapberry fruit cultivation is nearly 1 million tonnes through 1 million hectare of plantation. It is the one of the source for biodiesel production among the world^15^. In this investigation diesel, Biodiesel of Soapberry Seed and their 10% to 30% volume of blends (10BDSS, 20BDSS and 30BDSS) with diesel used in the CRDI engine for the identification of the better performance results based Biodiesel of Soapberry Seed blend with diesel, which have lesser emission results.

## Experimental procedure

**Figure 1:**
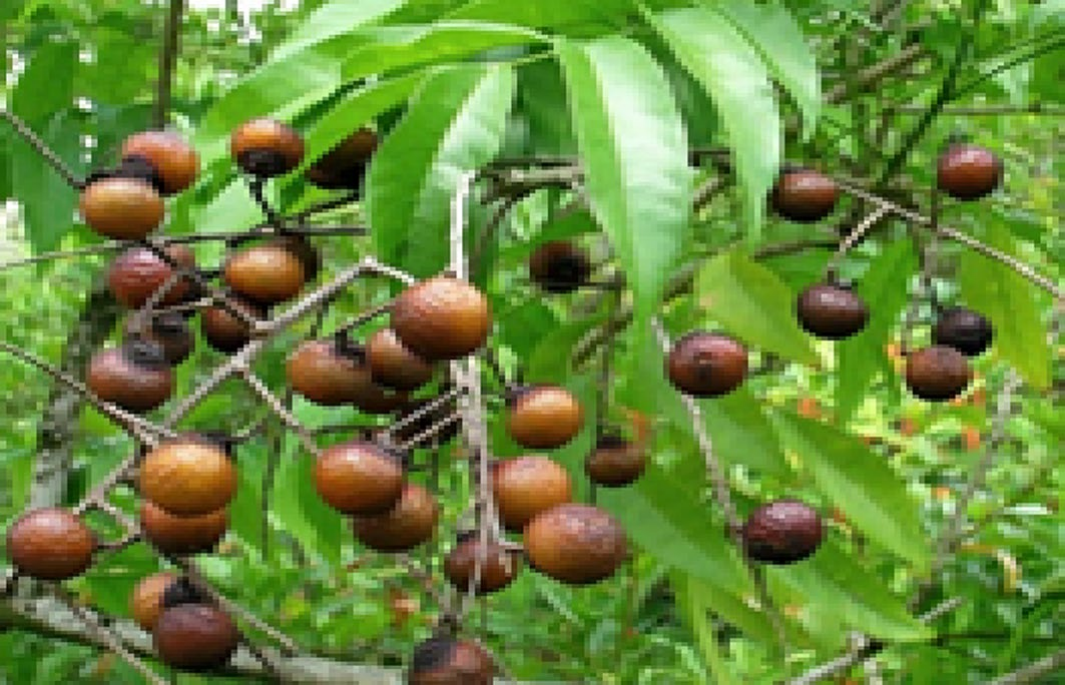
Soapberry.

Soapberry seeds (Fig. 1) purchased in Chennai and dried in sunshine for 10 days. The oil extracted by the tradition oil extraction such as cold press method. 72 kg of seed produce 32 lit of oil. This raw oil used to produce biodiesel by the transesterification process with methanol and KOH as a catalyst in the container with magnetic stirrer for 3 h with 65 °C of operating temperature. Then the 80% of biodiesel (BDSS—Biodiesel of Soapberry Seed) yield separated from glycerine after one day of cooling. This oil is 10–30% with 10% of incrementally blended with diesel by volume for this investigation with CRDI engine. 10BDSS is blend of 10%BDSS and 90% of diesel, 20BDSS is blend of 20%BDSS and 80% of diesel, and 30BDSS is blend of 30%BDSS and 70% of diesel. Properties details of blending mentioned in Table 1.

**Table 1:** Blending fuel properties.

Standards ASTM	D613	D6751	D93	D1298	D 445
Properties	Cetane index	Lower calorific value (kJ/kg)	Flash point (°C)	Density at 15 °C (kg/m^3^)	Viscosity at 40 °C (mm^2^/s)
Diesel	52	42000	51	832	3.41
100BDSS	55	37821	157	867	3.58
10BDSS	53.3	41582	61.6	835.5	3.427
20BDSS	52.6	41164	72.2	839	3.444
30BDSS	52.9	40746	82.8	842.5	3.461

**Figure 2:**
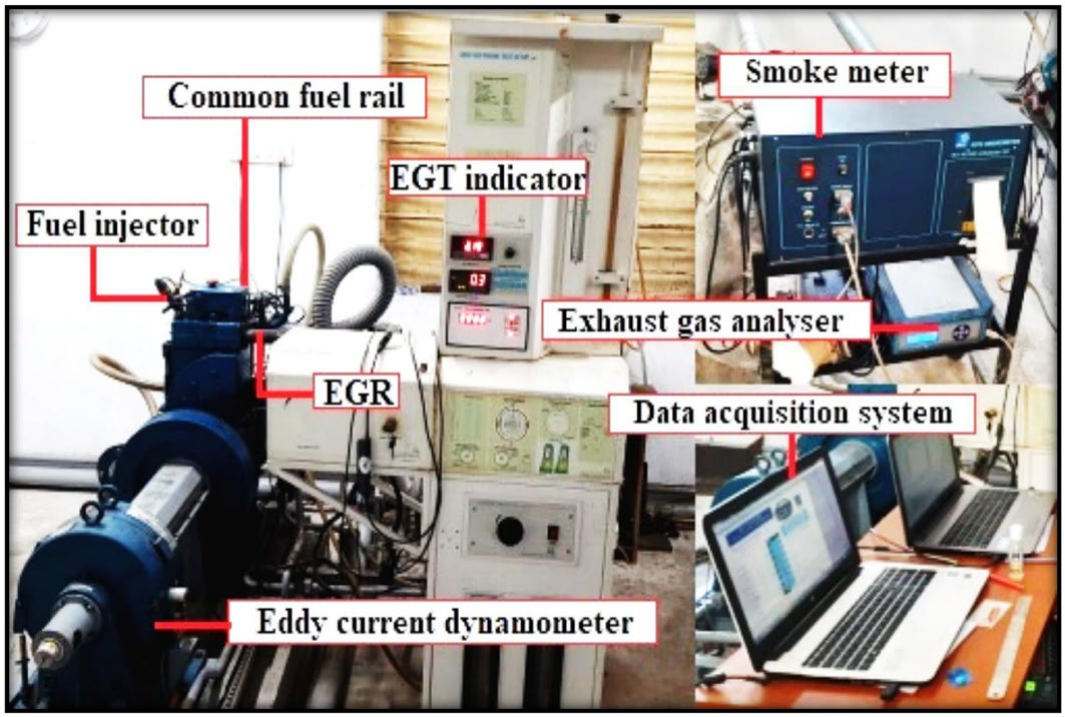
Experimental arrangements.

Figure 2 give the details about the experimental arrangement. It is the 1500 rpm 3.5 kW powered CRDI CI engine (Model: AV1—Kirloskar, 0.553 cubic capacity, water cooling) with variable compression ratio on single cylinder with injection pressure of 600 bar (Fuel injector of six hole controlled by solenoid). Colling done with water. Electronic injection done on Nira i7R (open electronic control unit) which collect the fuel pressure and flow data. Then the common arrangements like emission and smoke measurements done with Gas analyzer and smoke meter both are AVL products. Pressure of fuel injection maintained by the connected common rail for the fuel injection from the tank of fuel. Pressure transducer (0–5000 psi range) used to measure the in-cylinder pressure. Crank angle encoder (0–360 degree) used to measure the crank angel of rotation. All the date from the encoder, pressure transduce, fuel flow sensor, airflow sensor, injection pressure details were connected with the data equitation system and the computer. Engine run after complete arrangement with clear flawless observation. The individual fuels mentioned in the Table 1 used in this experimental arrangement for the Performance results.

## Results and discussion

### Combustion study

**Figure 3:**
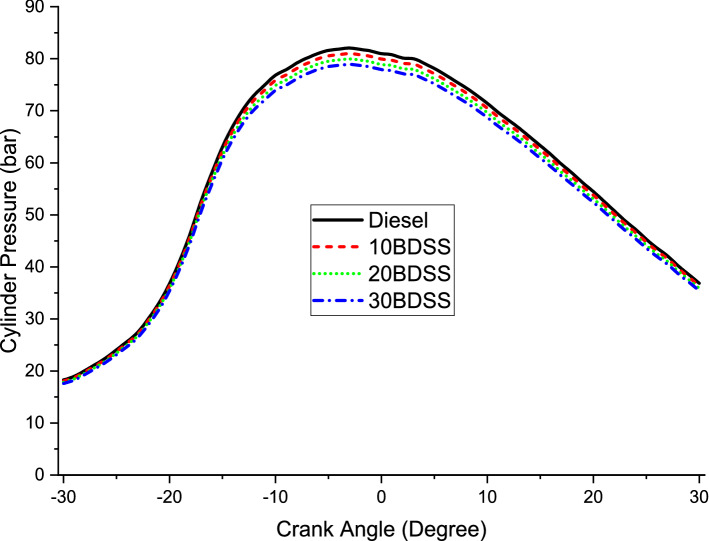
Cylinder Pressure differences on blending.

In this combustion studies explained about the in cylinder pressure variations and the heat release rate variation on the engine during the operation at highest load with different biodiesel blends and diesel. From the Fig. 3 mentioned that the maximum pressure obtained at maximum load on the cylinder of 78.93 bar, 79.99 bar, 81 bar and 81.96 bar by 30BDSS, 20BDSS, 10BDSS and Diesel. Here increasing pressure based order maintained to mention the in cylinder pressure. Diesel have the highest peak pressure.

The individual biodiesel and their blends have lesser peak pressure than diesel. At maximum load, the fuel consumption is more, which leads to increase in the gas pressure on the cylinder^16^. Individual diesel have 42,000 kJ/kg of calorific value. This is highest calorific value on the fuels properties comparison. So this lead highest peak pressure during operation^17^. The biodiesel of soapberry seed have lesser evaporation and reduced evaporation than diesel because of their lesser calorific value and higher density. This will leads on the variations of the cylinder pressure^18^.

**Figure 4:**
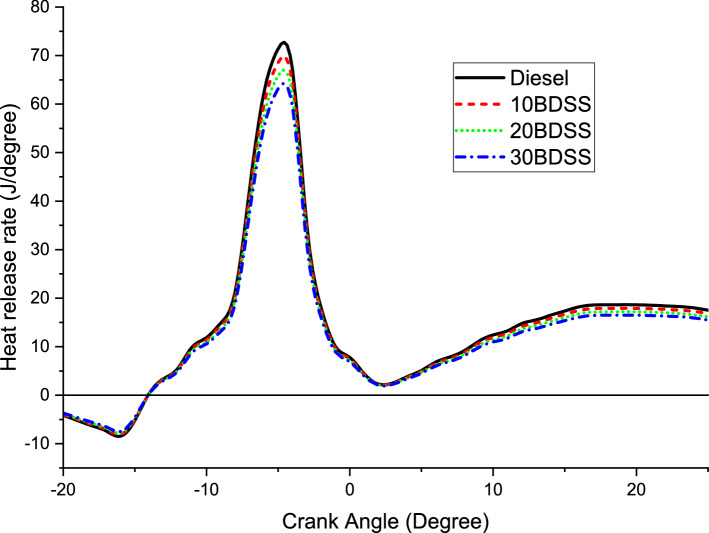
Influence of biodiesel blends in heat release rate.

The influence of the biodiesel blends in heat release rates obviously point out in the Fig. 4. Here, 72.25 J/deg of higher heat release rate obtained by the diesel. 10BDSS blend have 68.32 J/deg, 20BDSS fuel have 65.06 J/deg, and 30BDSS fuel have 61.15 J/deg of heat release rate at greatest load. Lesser contribution of the biodiesel of soapberry seed in blending have higher heat release rate vice versa. The oxygen content available on the biodiesel of soapberry seed leads to reduction on the higher heat release rate^19^. The lowest heat relkease rate gained by the 30% of biodiesel blend. Because of biodiesel of soapberry seed blend’s higher density and viscosity are influence the combustion characteristics. Therefore, it lead to decrease the heat release rate^20^.

### Performance analysis

In this performance analysis explained about the brake thermal efficiency and brake specific energy consumption variation on the engine during the various load condition with different biodiesel blends and diesel.

**Figure 5:**
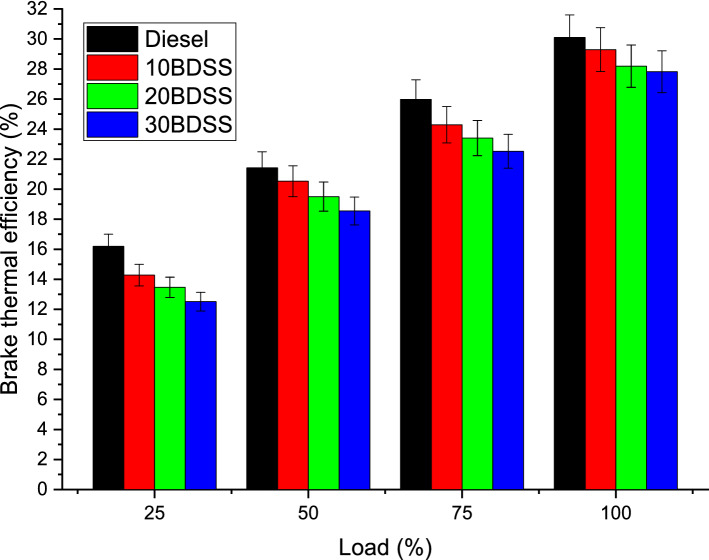
Influence of biodiesel blends in BTE (Brake thermal efficiency).

At 75% of load condition diesel have 25.98%, 10BDSS blend have 24.29%, 20BDSS fuel have 23.41%, and 30BDSS fuel have 22.52% of brake thermal efficiency. At full load, diesel have 30.10%, 10BDSS blend have 29.29%, 20BDSS fuel have 28.19%, and 30BDSS fuel have 27.82% of brake thermal efficiency. These brake thermal efficiency variations were mentioned in the Fig. 5. Efficiency increased with loads. Because of their calorific values of the blending, they produced lesser brake thermal efficiency when compared with diesel^21^. BDSS have more fatty acid^22^, Maximum participation of the biodiesel provide more reduction on the efficiency due to lesser viscosity, content of energy, density which lead to influence the evaporation of the fuel available in the cylinder during combustion^11,23^.

**Figure 6:**
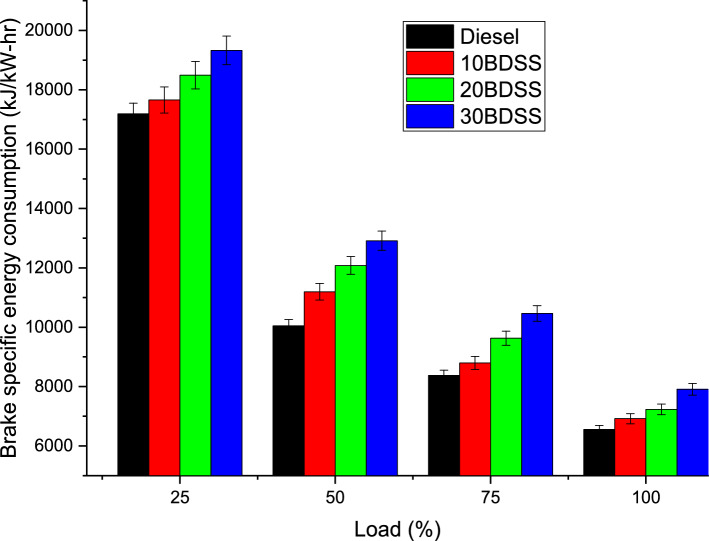
Influence of biodiesel blends in BSEC (Brake specific energy consumption).

Figure 6 describe about the brake specific energy consumption variations on the engine with different biodiesel blends at different loads. At 75% of load condition diesel have 8377.4 kJ/kW-hr, 10BDSS blend have 8794.49 kJ/kW-hr, 20BDSS fuel have 9628.67 kJ/kW-hr, and 30BDSS fuel have 10,462.8 kJ/kW-hr of brake specific energy consumption. Similarly at highest load, diesel have 6552.64 kJ/kW-hr, 10BDSS blend have 6917.59 kJ/kW-hr, 20BDSS fuel have 7230.41 kJ/kW-hr, and 30BDSS fuel have 7908.18 kJ/kW-hr of brake specific energy consumption.

Diesel have the lowest brake specific energy consumption than blends. This follows the common trends of the brake specific energy consumption such as load and brake specific energy consumption are inversely proportional^24^. These difference created by the lesser calorific values of the biodiesel of soapberry seed also higher amount of the fuel required to develop the similar amount of power^22,25^.

### Emissions analysis

In this emission analysis explained about the NOx emission, smoke opacity, carbon monoxide emission and hydro carbon emission on the engine during the operation at varieties of load with different biodiesel blends and diesel.

**Figure 7:**
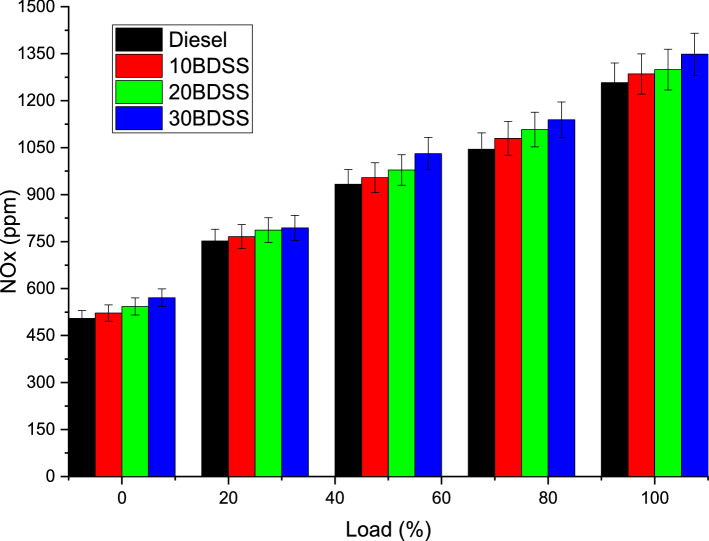
Influence of biodiesel blends in NOx emissions.

Figure 7 clearly express the influence of biodiesel blends for the NOx emission The temperature on the cylinder and supplied oxygen to the cylinder are the preferable response for the NOx emission. At 75% of load condition diesel have 1045 ppm, 10BDSS blend have 1080 ppm, 20BDSS fuel have 1108 ppm, and 30BDSS fuel have 1139 ppm of NOx emission produced. In the same way at 100% of load condition diesel have 1258 ppm, 10BDSS blend have 1286 ppm, 20BDSS fuel have 1300 ppm, and 30BDSS fuel have 1348 ppm of NOx emission produced. 30BDSS fuel have produced more NOx than other fuels because of the higher oxygen content available on the fuel. At higher loads increase in the pressure leads to the increase in the combustion temperature^23^. During the phase of premixed combustion, more heat generated cause the more NOx emission^16,26^.

**Figure 8:**
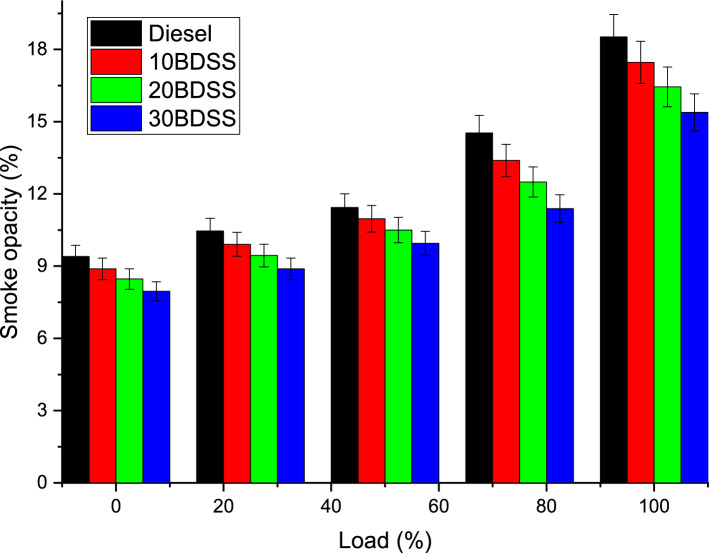
Influence of biodiesel blends in smoke opacity.

The major influence on the smoke opacity by the various biodiesel blends and diesel clearly mentioned in the Fig. 8. At 75% of load condition diesel have 14.53%, 10BDSS blend have 13.39%, 20BDSS fuel have 12.50%, and 30BDSS fuel have 11.39% of smoke opacity. At full load, diesel have 18.52%, 10BDSS blend have 17.46%, 20BDSS fuel have 16.44%, and 30BDSS fuel have 15.38% of smoke opacity. From these observation produced that the smoke opacity have reductions at lesser loads and rises gradually in maximum loads. At greater load, fuel supplied to the system is higher which create rich mixture in the chamber, these cause the smoke in the tail pipe^21^. Because of the oxygen content supremacy in the 30BDSS blend produce healthier combustion; these will reduce the smoke opacity than other considered fuels^10,27^.

**Figure 9:**
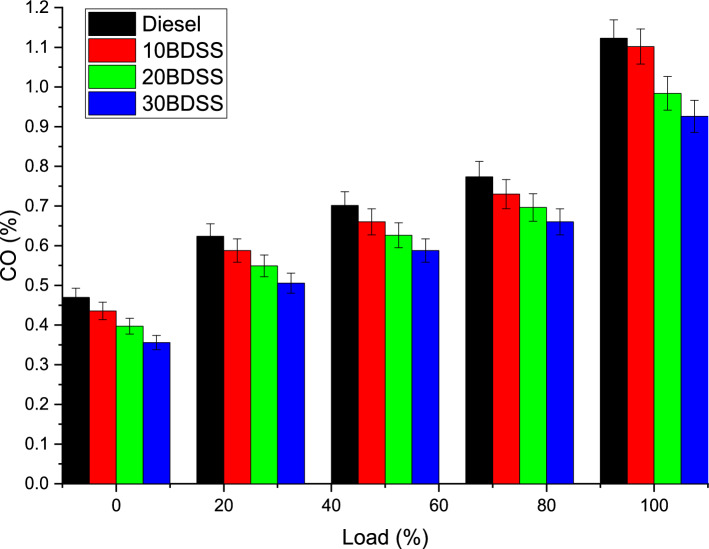
Influence of biodiesel blends in CO emissions.

The most important effect on the CO emission through the different biodiesel blends and diesel at different load clearly mentioned in the Fig. 9. At 75% of load condition diesel have 0.77%, 10BDSS blend have 0.73%, 20BDSS fuel have 0.70%, and 30BDSS fuel have 0.66% of CO emission. Similarly at 100% of load condition diesel have 0.92%, 10BDSS blend have 0.89%, 20BDSS fuel have 0.84%, and 30BDSS fuel have 0.81% of CO emission. These CO emission created by the incomplete combustion by reason of higher viscosity, spray penetration in untimely, temperature inside the cylinder, applied fuel involvement and the ratio of the hydrocarbon involved in the combustion^28^. Blending have lesser CO emission. Especially 30BDSS have lowest CO emission than other fuels at highest load. These all are because of the temperature of the combustion, incomplete mass burnt rate in maximum load^22,29^.

**Figure 10:**
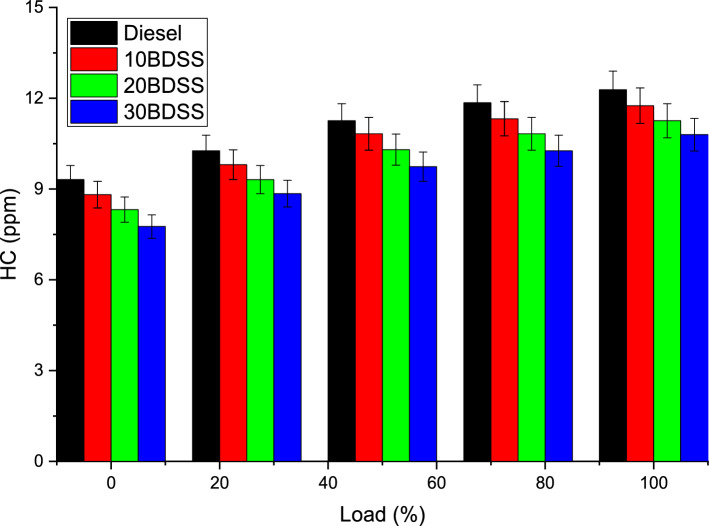
Influence of biodiesel blends in HC emissions.

Figure 10 noticeably express the influence of biodiesel blends for the HC emission. At 75% of load condition diesel have 12 ppm, 10BDSS blend have 11 ppm, 20BDSS fuel have 11 ppm, and 30BDSS fuel have 10 ppm of HC emission produced. In the same way at 100% of load condition diesel have 13 ppm, 10BDSS blend have 12 ppm, 20BDSS fuel have 12 ppm, and 30BDSS fuel have 11 ppm of HC emission produced. Increase in load lead to decrease in HC emission through the enhancing of the combustion efficiency with load augmentation. 30BDSS blend have lower HC emission than other fuels in all loads. The presence of more biodiesel not much affect the HC emission in tail pipe because of the better air fuel ratio obtained during the combustion in combustion chamber at the time of combustion^30^. Higher fatty acid^15^ contain biodiesel have more oxygen content it help to improve the combustion with lesser HC emission^23^.

## Conclusions

The blending of the biodiesel of soapberry seed (10% to 30%) produced from the transesterification process used CRDI engine at different load condition provide following as conclusions.

- Blending of the biodiesel of soapberry seed usage in the CRDI engine is possible at different loads. The percentage of biodiesel increase create the significant influence on the experimental results in combustion, performance and emission.
- Diesel have maximum brake thermal efficiency (30.10%) than other blends.
- The 30% biodiesel of soapberry seed (30BDSS) used blend have lowest peak cylinder pressure, lowest heat release rate in combustion than diesel.
- The 30% biodiesel of soapberry seed (30BDSS) used blend have lowest HC, CO and smoke emissions but NOx emission increased.
- The 30BDSS blend created uppermost NOx emissions than other fuels.
- 30BDSS have 27.82% of BTE, 1348 ppm of NOx emission, 78.93 bar of peak pressure, 61.15 J/deg of HRR with lesser CO (0.81%), HC (11 ppm) and smoke opacity (15.38%) emissions.
- It recommended that the utilization of 30% biodiesel of soapberry seed blend usage in CRDI engine without any modification have nearer performance and lesser emissions than diesel except NOx.

## Data Availability

The datasets generated during and/or analysed during the current study are available from the corresponding author and can be shared on reasonable request.
